# A Decrease in Hemodynamic Response in the Right Postcentral Cortex Is Associated With Treatment-Resistant Auditory Verbal Hallucinations in Schizophrenia: An NIRS Study

**DOI:** 10.3389/fnins.2022.865738

**Published:** 2022-05-25

**Authors:** Nana Liang, Sha Liu, Xinrong Li, Dan Wen, Qiqi Li, Yujie Tong, Yong Xu

**Affiliations:** ^1^Department of Psychiatry, First Hospital/First Clinical Medical College of Shanxi Medical University, Taiyuan, China; ^2^Shanxi Key Laboratory of Artificial Intelligence Assisted Diagnosis and Treatment for Mental Disorders, First Hospital of Shanxi Medical University, Taiyuan, China; ^3^Department of Mental Health, Shanxi Medical University, Taiyuan, China

**Keywords:** schizophrenia, auditory verbal hallucinations, treatment-resistant, near-infrared spectroscopy, verbal fluency task, hemodynamic response

## Abstract

**Background:**

Treatment-resistant auditory verbal hallucinations (TRAVHs) might cause an increased risk of violence, suicide, and hospitalization in patients with schizophrenia (SCZ). Although neuroimaging studies have identified the neural correlation to the symptom of AVH, functional brain activity that correlates particularly in patients with TRAVH remains limited. Functional near-infrared spectroscopy (fNIRS) is a portable and suitable measurement, particularly in exploring brain activation during related tasks. Hence, our researchers aimed to explore the differences in the cerebral hemodynamic function in SCZ-TRAVH, patients with schizophrenia without AVH (SCZ-nAVH), and healthy controls (HCs), to examine neural abnormalities associated more specifically with TRAVH.

**Methods:**

A 52-channel functional near-infrared spectroscopy system was used to monitor hemodynamic changes in patients with SCZ-TRAVH (*n* = 38), patients with SCZ-nAVH (*n* = 35), and HC (*n* = 30) during a verbal fluency task (VFT). VFT performance, clinical history, and symptom severity were also noted. The original fNIRS data were analyzed using MATLAB to obtain the β values (the brain cortical activity response during the VFT task period); these were used to calculate Δβ (VFT β minus baseline β), which represents the degree of change in oxygenated hemoglobin caused by VFT task.

**Result:**

Our results showed that there were significant differences in Δβ values among the three groups at 26 channels (ch4, ch13-15, 18, 22, ch25–29, 32, ch35–39, ch43–51, F = 1.70 to 19.10, *p* < 0.043, FDR-corrected) distributed over the prefrontal–temporal cortical regions. The further pairwise comparisons showed that the Δβ values of 24 channels (ch13–15, 18, 22, 25, ch26–29, ch35–39, ch43–49, ch50–51) were significantly lower in the SCZ group (SCZ-TRAVH and/or SCZ-nAVH) than in the HC group (*p* < 0.026, FDR-corrected). Additionally, the abnormal activation in the ch22 of right postcentral gyrus was correlated, in turn, with severity of TRAVH.

**Conclusion:**

Our findings indicate that specific regions of the prefrontal cortex may be associated with TRAVH, which may have implications for early intervention for psychosis.

## Introduction

Auditory verbal hallucinations (AVHs) are characterized as the experiences of hearing a voice or any other sounds for languages under the conditions of conscious state with no external stimulus (McCarthy-Jones and Resnick, 2014; Upthegrove et al., 2016). As the most prominent and burdensome symptoms of schizophrenia (SCZ), the prevalence of AVH reported rates of 60–80% (Alderson-Day et al., 2015). However, roughly 30% of patients with AVH [treatment-resistant auditory verbal hallucinations, (TRAVHs)] are not responded well for current clinical treatments (Kennedy et al., 2014) and might cause an increased risk of violence, suicide, and hospitalization (Upthegrove et al., 2016; Dugré et al., 2018). From 2013 to 2018, the International Consortium on Hallucination Research (ICHR) suggested that clinical and pathological features of AVH should be explored from all perspectives (Waters, 2012; Jardri et al., 2019).

Currently, the underlying pathological mechanisms remain poorly understood, but an array of studies have explored the neural correlations of AVH. For instance, the functional neuroimaging evidence explored the various brain areas related to AVH, some of which were involved in inner speech generation, inner speech perception, and hallucinations processing (Stephane et al., 2001; Jardri et al., 2011; Kühn and Gallinat, 2012). According to cognitive neurophysiology, inner speech has played an important role in a series of theories of AVH in patients with SCZ (Shergill, 2003; Langland-Hassan, 2010; Wayne, 2012). One prominent hypothesis suggests that inner speech fails to be labeled as internally generated, and instead being perceived as an autonomous, non-self-voice (Jones, 2010). The neurological evidence of inner speech model involved in left inferior frontal gyrus, bilateral temporal cortex, the supplementary motor area, premotor cortex, precentral, postcentral gyri, inferior parietal lobe, right insula, and posterior cerebellar cortex bilaterally (Moseley et al., 2013; Curčić-Blake et al., 2017). Other results displayed that AVH may arise from the abnormal activation in the left superior temporal region and the left prefrontal cortex during the processing of inner speech (Allen et al., 2007; Simons et al., 2010; Jardri et al., 2011). These above research displayed the neural activation associated with AVH and inner speech were found to offer more convincing evidence for the disorder of inner speech in patients with AVH. Based on these findings, we hypothesized that functional abnormalities in the prefrontal and temporal cortex, as measured with functional near-infrared spectroscopy (fNIRS), underly TRAVH among patients with SCZ, supplying more evidence to have a better understanding of the inner speech model of AVH.

Functional near-infrared spectroscopy (fNIRS) (Herrmann et al., 2003) is a non-invasive imaging device to evaluate brain function, which has several advantages. fNIRS is easy to set up, requires minimal, constrains, and does not occupy a large space (Wei et al., 2020). Additionally, it is safe, nonrestrictive, quiet, and economical for measurement (Ferrari and Quaresima, 2012). fNIRS signals are believed to assess the neuronal activity according to a phenomenon known as neurovascular coupling (Gsell et al., 2000) and have strong correlations of blood oxygenation level-dependent (BOLD) signals measured by fMRI (Sasai et al., 2012). Moreover, fNIRS is less sensitive to motion artifacts and can be operated without the acoustic scanner noise, which is more suitable for evaluating patients with AVH during related tasks (Ehlis et al., 2014). fNIRS can monitor both oxy- and deoxy-hemoglobin levels in the cerebral cortex. Since the oxy changes are greater than deoxy-hemoglobin, oxy-hemoglobin is used as the brain activity marker (Ferrari and Quaresima, 2012), while the verbal fluency task (VFT) is considered as the sensitive indicators of language function and frontal and temporal lobe function, such as multiple high-level cognitive process, including executive function, attention, inhibition, and working memory, which has been proposed as an assessment measure of functional impairment and illness severity (Herrmann et al., 2003; Sasai et al., 2012; Koike et al., 2013). In addition, a large number of fNIRS studies have explored and compared the prefrontal cortex activation between patients with SCZ and healthy control (HC) by VFT. Previous findings showed that patients with SCZ had reduced oxy-hemoglobin in the bilateral prefrontal and temporal cortices during the VFT task (Suto et al., 2004; Takizawa et al., 2008, 2014; Koike et al., 2013, 2017; Kumar et al., 2017), which indicated that VFT is a widely accepted cognitive task for elucidating brain dysfunction related to psychotic state. Among patients with SCZ, the symptom of AVH was associated with the decreases in the cerebral blood flow in the brain regions, including postcentral gyri (inner speech production) (Tremblay et al., 2003) and right supplementary motor area (inner speech imagery) (McGuire et al., 1995; Raij and Riekki, 2012), as well as the bilateral dorsolateral prefrontal cortices (inner speech monitoring) (Cui et al., 2017). The gray matter density reduction in right postcentral gyri was found to be specifically negatively related to the severity of AVH(García-Martí et al., 2008). Whereas, such studies were argued to favor that patients with SCZ with AVH and without AVH showed significantly decreased ReHo in the bilateral postcentral gyrus (Zhuo et al., 2016), based on the different results, it is even more important to know whether the AVH-related brain region, such as postcentral gyri, is the common neural substance underlying both the inner speech and AVH. Specifically, for SCZ-TRAVH, clarification of neural mechanisms is of great clinical significance for treatment. Therefore, in the present study, we utilized multi-channel fNIRS to investigate whether the symptom of TRAVH is associated with functional alterations in the brains of patients with SCZ during VFT and the underlying neurological mechanisms for the inner speech related to AVH.

## Method

### Participants

A total of one hundred and three participants (≥18 years old) were included in this study. Patients with TRAVH (*n* = 38 participants) had experienced persecutory voices at least 12 months and not respond to at least two anti-psychotic trials of anti-psychotic drugs at equivalent doses to 600 mg/day of chlorpromazine. Patients with SCZ without AVH (SCZ-nAVH) (*n* = 35 participants) did not experience AVH or were fully remitted from AVH in the past year. All of the patients completely satisfied the DSM-V diagnostic criteria for SCZ, wherein the structured clinical interview for DSM-V (SCI-D) was conducted by two experienced psychiatrists. Exclusion criteria were as follows: (1) any change in dose of anti-psychotic drugs in recent 3 months; (2) electroconvulsive therapy within the past 6 months; (3) alcohol or substance disorder; (4) traumatic brain injury; and (5) neurological impairment or intellectual disability. HC (*n* = 30) reported no past or current of Axis I or II disorders, and no history of Axis I disorder in first- or second-degree family members. HCs were excluded if they had the following conditions: (1) organic disease; (2) alcohol abuse or drug dependence; (3) received psychotherapy in the past; and (4) other conditions that disqualified the subject from the study, as determined by the investigators. For patients with SCZ, we used Positive and Negative Syndrome Scale (PANSS) (Lewine et al., 1983) to evaluate clinical symptoms. The levels of functioning were evaluated using the modified Global Assessment of Functioning (GAF) (Asahi et al., 2004; Okada et al., 2016). The severity of the AVH was quantified by the scale of The Psychotic Symptom Rating Scales, auditory hallucinations (PSYRATS-AH) (Haddock et al., 1999) and Positive and Negative Syndrome Scale, hallucinations item (PANSS-P3). This study has been approved by the Ethics Committee of First Hospital of Shanxi Medical University (K047). Before participation in the study, all the participants were able to read, understand the consent form, and signed the informed consent.

### Verbal Fluency Task

We adopted a Chinese version of phonological VFT (Wei et al., 2020). This measurement was taken under a quiet environment. Participants were asked to remain seated with their eyes open, avoid excessive body, minimize head movements, and focus on a cross-displayed during the measurements. It comprised a 30-s pre-task period, a 60-s task period, and a 70-s post-task period. During the pre- and post-task periods, the participants were asked to constantly say “1, 2, 3, 4, 5” repeatedly. During the task period, the participants were asked to generate as many four-character idioms or phrases as possible, which begin with the designated Chinese characters (such as, “大,” “白,” and “天,” indicating big, white, and sky, respectively). There was a total of three cue characters that were changed every 20 s during the 60-s task period. The number of unique words was recorded as the VFT task performance.

### NIRS Measurement

A 52-channel fNIRS system (ETG-4100. Hitachi Medical Co., Tokyo, Japan) uses 2 NIR light wavelengths (695 and 830 nm) to measure the hemodynamic responses in the prefrontal cortices and superior temporal cortices (FOIRE-4100, Hitachi Medical Co, Japan). This system had 16 light detectors and 17 light emitters, all of which were arranged in a 3 × 11 array to form 52 measurement channels according to the international 10–20 system. This arrangement allowed for hemodynamic response covered mainly in the entire bilateral prefrontal cortices, and the anterior and superior parts of the temporal cortex to be measured.

### NIRS Signal Analysis

The near-infrared spectroscopy signals were processed with the NIRS-SPM toolbox (Jang et al., 2009; Ye et al., 2009), which is a MATLAB-based software package for statistical analysis. NIRS_SPM is based on the general linear model (GLM) method of data analysis. The GLM method is a well-established regression method widely used for fMRI data analysis and has also been applied in the analysis of fNIRS data. The GLM includes the variables Y, X, β, and ε, as follows: Y = βX + ε. Y represents the data actually detected by fNIRS, consisting of the data matrix containing time series and detection channels. X is the predicted value based on the experimental design; this value is obtained by convolving the hemodynamic response function with the event sequence. β is the coefficient of fit, which reflects how much of the Y signal is caused by the test matrix X. In this study, it represents the level of brain cortical functional activity response caused by VFT. Finally, ε represents unexplained errors. fNIRS raw data were conducted using a hemodynamic response function (HRF) and discrete cosine transform (DCT) to remove noise, such as the drift and artificial noises (such as head motion) (Brigadoi et al., 2014). According to the previous studies, compared with the changes in deoxygenated hemoglobin concentrations, oxygenated hemoglobin concentrations were the more sensitive and reliable measures of fNIRS used for later analysis (Strangman et al., 2003).

### Statistical Analysis

The Δβ value of oxy-hemoglobin (VFT β value minus baseline β value) was used to assess the degree of activation of the brain cortex caused during VFT task period and analyzed using SPSS v22.0 (SPSS Inc, Chicago, IL, USA). The data of categorical variables were performed using the chi-square test. One-way analysis of variance (ANOVA) with Bonferroni corrected *post hoc* pairwise comparisons was then used to determine the effect of different groups on continuous variables. For channels that exhibit significant differences in the *post hoc* Bonferroni corrected test, Pearson's correlation coefficient was performed to determine the relationship between the Δβ value of oxy-hemoglobin and number of words, PANSS-positive score, -negative score, general psychopathology score, P3 score, GAF, and PSYRATS-AH score. Then, multiple regression analyses were conducted using changes in oxy-hemoglobin as the dependent variable and age, sex (men = 1, women = 2), the number of words, PSYRATS-AH scores, PANSS subscores, P3 sores, and GAF scores as the independent variables. All tests were two-tailed, and the obtained *p*-value was corrected by false discovery rate (FDR) (Benjamini et al., 2001; Singh and Dan, 2006). P(FDR) < 0.05 was considered statistically significant.

## Results

### Demographic, Clinical, and Psychosocial Characteristics of All the Participants

This study included 38 patients with SCZ-TRAVH, 35 patients with SCZ-nAVH, and 30 HCs. Table 1 summarizes the samples of sociodemographic and clinical data. The three different groups did not differ in gender, age, education years, illness duration, number of words, PANSS score, PANSS subscore, and GAF score. PSYRATS-AH and PANSS P3 scores were significantly higher in the SCZ-TRAVH group than in the SCZ-nAVH group (all *p* < 0.001) (Table 1).

**Table 1:** Demographic, clinical, and psychosocial characteristics of all participants (*n* = 103).

**Variable**	**SCZ-TRAVH**	**SCZ-nAVH**	**HC**	***P***
Gender	38	35	30	0.879
Male	20	17	14	
Female	18	18	16	
Age, years	29.89 ± 6.22	29.54 ± 6.43	29.03 ± 6.67	0.857
Education, years	13.17 ± 2.99	13.76 ± 2.68	14.04 ± 1.86	0.210
Duration of illness (years)	4.10 ± 1.00	4.80 ± 0.90	NA	0.095
Number of words	7.47 ± 1.26	7.63 ± 1.48	8.03 ± 1.35	0.631
PANSS total score	73.37 ± 13.07	71.97 ± 12.15	NA	0.639
PANSS-positive score	20.92 ± 5.48	19.49 ± 5.83	NA	0.282
PANSS-negative score	20.82 ± 6.22	20.06 ± 6.89	NA	0.632
PANSS general psychopathology Score	31.63 ± 8.99	32.43 ± 8.06	NA	0.692
P3 score	4.29 ± 0.65	1.03 ± 0.17	NA	<0.001
PSYRATS-AH score	26.39 ± 4.58	0.06 ± 0.24	NA	<0.001
GAF score	47.03 ± 8.97	49.66 ± 10.54		0.253

*All subjects were Han ethnicity, right hand, with no religious affiliation*.

*Data represent number and mean ± standard deviation. PANSS, Positive and Negative Scale; PSYRATS-AH, The Psychotic Symptom Rating Scales, auditory hallucinations subscale; P3, hallucinations; GAF, the modified Global Assessment of Functioning*.

### Hemodynamic Response During the VFT

**Figure 1:**
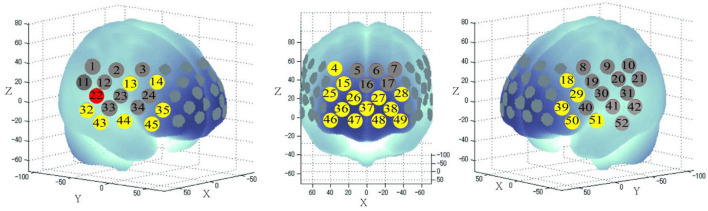
Three-dimensional cerebral maps of oxy-hemoglobin response patterns during the task periods. The red and yellow colors of 26 channels exhibit significant changes in meanΔβvalues of oxy-hemoglobin during the VFT task period among the three groups, as determined using one-way ANOVA tests (FDR-corrected *p* < 0.05).

**Figure 2:**
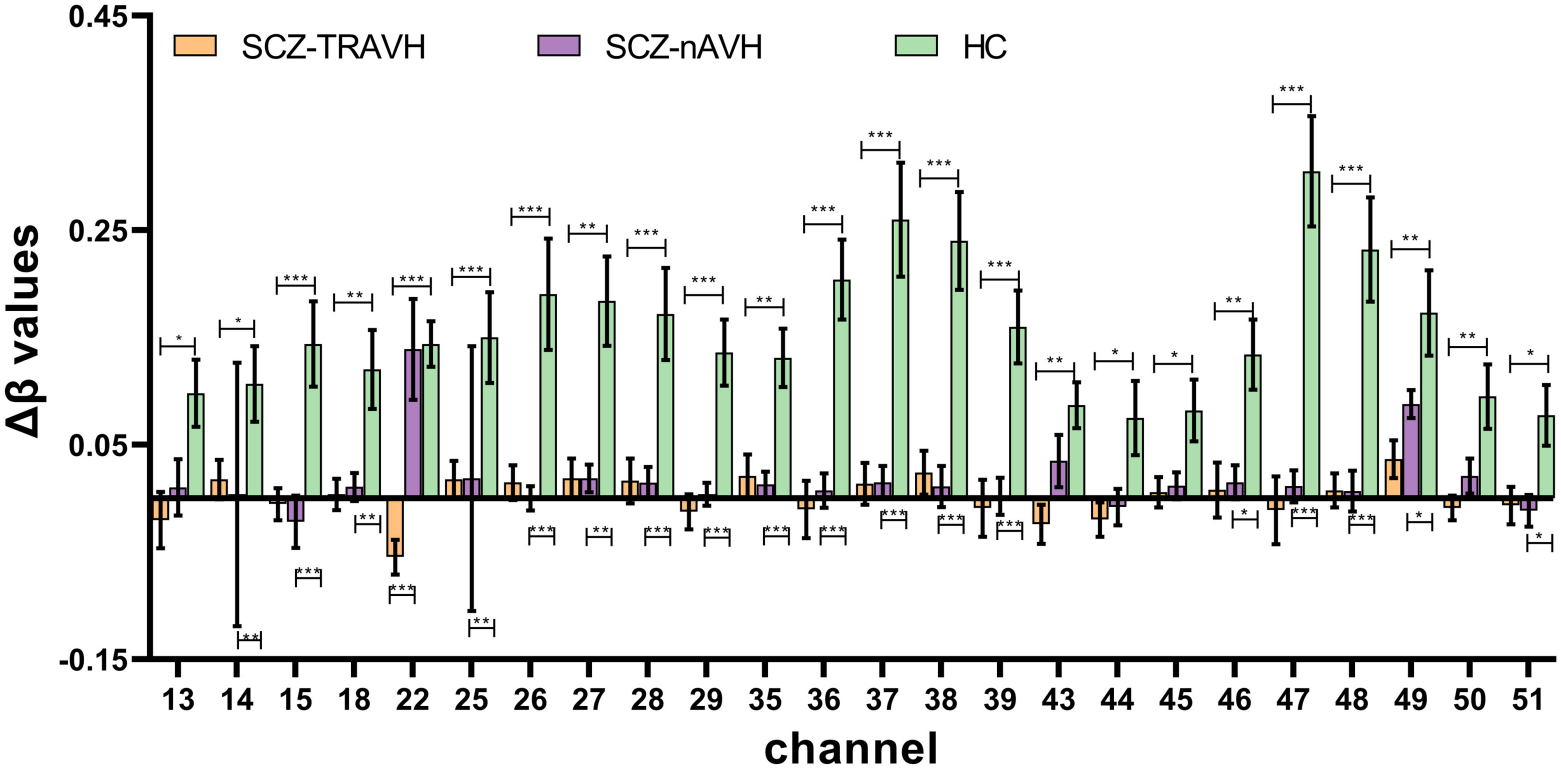
Bonferroni corrected *post hoc* pairwise comparison analysis results show significant difference in meanΔβvalues of oxy-hemoglobin on 24 channels (ch13–15, ch18, ch22, ch25–29, ch35–39, ch43–51). Error bars represent standard errors (all FDR-corrected *p < 0.05; **p < 0.01; ***p < 0.001; SCZ-TRAVH and/or SCZ-nAVH vs. HC group).

One-way repeated-measures ANOVA showed that there were significant differences in Δβ values among the three groups at 26 channels (ch4, ch13–15, 18, 22, ch25–29, 32, ch35–39, ch43–51, *F* = 1.70 to 19.10, *p* < 0.043, FDR-corrected, Figure 1) distributed over the prefrontal–temporal cortical regions, whereas there were no statistically significant differences in any of the other channels. The Δβ values in the above channels were further compared between groups (using Bonferroni corrected *post hoc* pairwise comparisons). The further comparison results showed that the Δβ values of 24 channels (ch13–15, 18, 22, 25, ch26–29, ch35–39, ch43–49, ch50–51) were significantly lower in the schizophrenia group (SCZ-TRAVH and/or SCZ-nAVH) than in the HC group (*p* < 0.026, FDR-corrected, Figure 2). In addition, the SCZ-TRAVH group has a lower hemodynamic response at channel 22 of the right postcentral gyrus compared to the SCZ-nAVH group (*p* = 0.0029; Figure 2) and HC group (*p* = 0.0029; Figure 2), respectively, whereas no significant difference was observed in this channel between SCZ-nAVH group and HC group.

### Correlation Between Demographic Characters and FNIRS Variables

**Figure 3:**
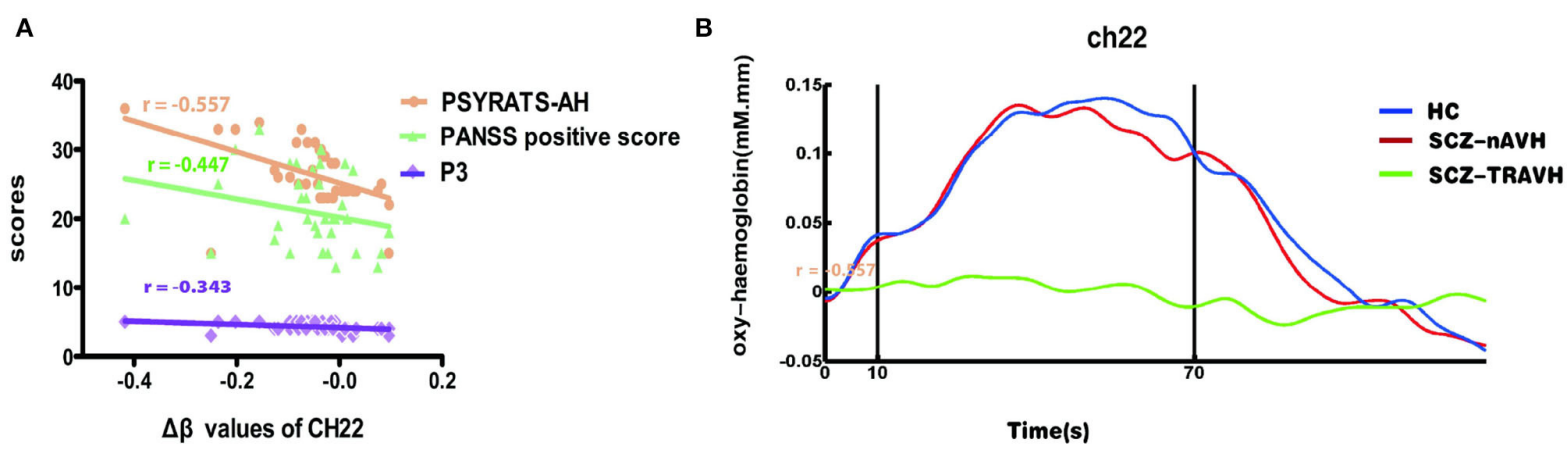
**(A)** The Δβ values of oxy-hemoglobin were significantly associated with severity of hallucinations on the channel 22, including PSYRATS-AH, PANSS-positive score, and P3 score. **(B)** Average oxy-hemoglobin waveforms of three groups at the cortical regions. Vertical lines demarcate the start and end of the VFT task period.

Within the SCZ-TRAVH group, we observed trend-level negative correlations between the Δβ values of oxy-hemoglobin of the ch22 and P3 score (*r* = −0.343, *p* = 0.035, FDR-uncorrected). Similarly, a significant association was observed between Δβ values of oxy-hemoglobin at channel 22 and the score of the PSYRATS-AH scales (*r* = −0.577, *p* < 0.001). Also, a slight negative correlation was found between the PANSS-positive score and the Δβ values of oxy-hemoglobin at channel 22 (*r* = −0.440, *p* = 0.006). No other significant correlations were found between Δβ values of oxy-hemoglobin at channel 22 and score (PANSS-negative score, PANSS general psychopathology score, GAF scores, and the number of words). The association between the Δβ values of channel 22 and PSYRATS-AH remained significant after controlling for age, sex (men = 1, women= 2), the number of words, PANSS subscores, P3 sores, and GAF scores in a multiple regression analysis (*R*^2^ = 0.382, adjusted *R*^2^ = 0.212, β = −0.636, *t* = −2.272, *p* = 0.031) (Figure 3A). For SCZ-nAVH group, there was no significant association between score (PSYRATS-AH and PANSS subscores) and the change of fNIRS signals at channel 22 (all *p* > 0.05).

Nevertheless, the average waveforms of channel 22 of three groups illustrate that the increase in oxy-hemoglobin during the VFT task period was significantly higher in the patients with SCZ-nAVH and HC than in the patients with SCZ-TRAVH (Figure 3B). In addition, mean oxy-hemoglobin at channel 22 was associated with the symptom of AVH. Taken together, the results suggest that hemodynamic dysfunction in channel 22 seems to be associated with TRAVH.

## Discussion

This study used fNIRS to explore the association between hemodynamic response in the prefrontal and temporal cortices and TRAVH symptom in patients with SCZ. Our results indicated that patients with SCZ-TRAVH exhibited lower activation in the right postcentral gyrus than did those without the symptom of AVH.

First, in terms of oxy-hemoglobin, our study observed that the SCZ-TRAVH and SCZ-nAVH group had lower brain activation in extensive prefrontal and temporal cortices in contrast to the HC group, which were similar to the previous fNIRS studies showing the lower oxy-hemoglobin response in similar brain cortices during the related task (Takizawa et al., 2008, 2014; Wei et al., 2020). In addition, the brain activity of 24 channels in the SCZ-TRAVH group was lower than that in the HC group, whereas the oxy-hemodynamic response of only 18 channels in the SCZ-nAVH group was lower. According to the previous fMRI studies, hypoactivation of the bilateral prefrontal cortex and temporal cortices in patients with SCZ has been reported, which is associated with confusion of imagined items as perceptual events (Simons et al., 2006). Taken together, the results suggested that hemodynamic dysfunction in the prefrontal cortex and temporal cortices may be more extensive in patients with SCZ-TRAVH than patients without AVH.

In our result, SCZ-TRAVH group have lower hemodynamic response at channel 22 of the right postcentral gyrus compared to SCZ-nAVH group. This result was replicated in the fMRI study that patients with AVH showed decreased cerebral blood flow in the bilateral superior and postcentral gyri compared with patients with SCZ-nAVH (Cui et al., 2017). In addition, a significant negative association emerged between the Δβ value of oxy-hemoglobin in the region of ch22 and severity of AVH symptom [PSYRATS-AH score, PANSS-positive score, and P3 (hallucinations) score]. The association between Δβ values of channel 22 and PSYRATS-AH remained significant after controlling for other scales scores and VFT performance in a multiple regression analysis. This meaningful finding partially agreed with previous researches reported significant association between the reduction of bilateral postcentral gyrus volume and the severity AVH in SCZ (García-Martí et al., 2008; Nenadic et al., 2010). It was not difficult to find that among the above associated scales, PSYRATS-AH had the highest correlation, followed by PANSS-positive score. According to the consensus, PSYRATS-AH is a more detailed and multidimensional examination than PANSS and P3 for hearing voices, which had generally strong reliability and been widely used in diverse clinical studies of neural correlations of AVH (Ratcliff et al., 2011). Considering the highest correlation of PSYRATS-AH with CH22 signal changes, this may largely suggested associations of decreased activation in the postcentral gyrus with the putative mechanisms by which psychotic symptoms are generated, especially AVH. Additionally, the previous fMRI studies reported the right postcentral gyrus related to AVH and may be a new therapeutic target in the future (van Lutterveld et al., 2013). Additionally, Zui Narita et al.'s research further explored the potential utility of fNIRS for the prediction of effects of tDCS on psychotic symptoms (Narita et al., 2018). Although the findings show different potential therapeutic brain regions, these results support the concept that fNIRS may provide a valid method to evaluate brain functions based on blood flow, similar to fMRI (Lee et al., 2018).

The mechanism by which brain activity measured by fNIRS should be considered in relation to suffering from AVH symptoms. The postcentral gyrus regarded as one location of classically and primary defined somatosensory cortex (Skipper et al., 2005; Pérez-Bellido et al., 2018). A meta-analysis reflected the association of AVH with activation in the postcentral regions related to subvocal speech (Kühn and Gallinat, 2012) and therefore being considered involving inner speech generation (Feinberg, 1978; Shergill et al., 2002), while the symptoms of AVH were consistently associated with the disorders of generation of inner speech (inner verbal thoughts) (McGuire et al., 1996; DeLisi, 2001). According to other neuroimaging researches, inner speech-related brain region involved in the left inferior frontal, the right pre- and postcentral, and both superior temporal gyri (Shergill et al., 2002). Besides, another recent study further demonstrated that the postcentral gyrus was involved in the auditory information process and even auditory hallucination symptoms (Joo et al., 2020). It seemed that the inner speech generation played a role in the pathogenesis of AVH (McGuire et al., 1995). Taken together with the above neurological evidence, the decreased activation of postcentral gyrus may underlie the basis for disorders of inner speech generation and imply alterations in the monitoring of inner speech, leading to a higher risk for suffering from the symptom of AVH, partially consistent with inner speech model of AVH.

In this study, we focus on a limited group of SCZ-TRAVH whose AVH symptom was at a relatively stable phase to develop in-depth knowledge of pathological features of AVH and the heterogeneous nature of this disease. Additionally, we used fNIRS to reveal abnormal brain function during VFT task, which was corresponded to the resting-state and task-state fMRI studies' results (van Lutterveld et al., 2013; Cui et al., 2017), which make fNIRS as a suitable research tool to explore the underlying neural alterations of patients with TRAVH during related tasks. Our significant finding may promote the development in the design of the novel therapeutic strategies for TRAVH and encourage the application of fNIRS analysis to follow-up studies with a larger sample size to explore the underlying neuroimaging mechanism and understand the pathophysiological correlations of TRAVHs in SCZ.

Nevertheless, there are some clear limitations associated with our research. First, patient's sample sizes in this study were relatively small for aiming to offer a specific activation pattern for SCZ-TRAVH. Therefore, increasing the sample size might identify further brain regions associated with AVH. Second, although our researchers focused on studying a limited group of patients with SCZ with stable psycho-pathological features (AVH) resistant to drug treatment to obtain the activation difference in potential brain regions, we cannot fully rule out the medicinal effects. However, according to Koike et al.'s study (Koike et al., 2013), they emphasized that there is no association between fNIRS activity of patients with SCZ and doses of drugs. Furthermore, there was no significant difference in medication treatment between the SCZ-TRAVH and SCZ-nAVH groups in our study. In addition, we did not obtain data related to cognitive symptoms in our participants. Since cognitive symptoms are associated with the prefrontal dysfunction for patients with SCZ, further study with an assessment of cognitive symptoms is warranted.

The present multi-channel fNIRS study showed an association between TRAVH-induced hemodynamic impairments in the right postcentral gyrus and a history of TRAVH among patients with SCZ. These findings may help to provide evidence for the common theories regarding the “inner speech” and AVH, facilitating to do the design of novel therapeutic methods. A further study is remained to explore the long-term outcomes of SCZ-TRAVH through the fNIRS measurement.

## Conclusion

Our study used NIRS technology to explore and compare the abnormal activation difference in brain regions for patients with SCZ with TRAVH and nAVH. We found a negative association between the decreased activation of right postcentral gyrus and severity of TRAVH. Further, this study supplied a possible explanation for the occurrence of TRAVH in SCZ from the inner speech insights, which may have implications for an early biological evidence-based intervention for TRAVH.

## Data Availability Statement

The raw data supporting the conclusions of this article will be made available by the authors, without undue reservation. Requests to access these datasets should be directed to 761620286@qq.com.

## Ethics Statement

Written informed consent was obtained from the individual(s) for the publication of any potentially identifiable images or data included in this article.

## Author Contributions

NL, YT, and SL contributed to the study design and were involved in data acquisition, analysis, and interpretation. XL, DW, QL, and YX contributed to the study design and data interpretation. All authors participated in the drafting or critical review of the article, gave final approval of the version to be published, and agreed to be accountable for all aspects of the work.

## Funding

This work was supported by the National Natural Science Foundation of China (81971601 and 81571319), the National Key Research and Development Program of China (2016YFC1307004), the Wu Jieping Foundation (320.6750.18283), the special project for guiding the transformation of scientific and technological achievements in Shanxi Province (201904D131020), and the Multidisciplinary Team for Cognitive Impairment of Shanxi Science and Technology Innovation Training Team (201705D131027).

## Conflict of Interest

The authors declare that the research was conducted in the absence of any commercial or financial relationships that could be construed as a potential conflict of interest.

## Publisher's Note

All claims expressed in this article are solely those of the authors and do not necessarily represent those of their affiliated organizations, or those of the publisher, the editors and the reviewers. Any product that may be evaluated in this article, or claim that may be made by its manufacturer, is not guaranteed or endorsed by the publisher.

## References

[B1] Alderson-Day B. McCarthy-Jones S. Fernyhough C. (2015). Hearing voices in the resting brain: A review of intrinsic functional connectivity research on auditory verbal hallucinations. Neurosci. Biobehav. Rev. 55, 78–87. 10.1016/j.neubiorev.2015.04.016 25956256 PMC5901708

[B2] Allen P. Amaro E. Fu C. Williams S. Brammer M. Johns L.. (2007). Neural correlates of the misattribution of speech in schizophrenia. Br. J. Psychiatry. 190, 162–169. 10.1192/bjp.bp.106.025700 17267934

[B3] Asahi S. Okamoto Y. Okada G. Yamawaki S. Yokota N. (2004). Negative correlation between right prefrontal activity during response inhibition and impulsiveness: A fmri study. Eur. Arch. Psychiatry Clin. Neurosci. 254, 245–251. 10.1007/s00406-004-0488-z 15309395

[B4] Benjamini Y. Dan D. Elmer G. Kafkafi N. Golani I. (2001). Controlling the false discovery rate in behavior genetics research. Behav. Brain Res. 125, 279–284. 10.1016/S0166-4328(01)00297-2 11682119

[B5] Brigadoi S. Ceccherini L. Cutini S. Scarpa F. Scatturin P. Selb J.. (2014). Motion artifacts in functional near-infrared spectroscopy: a comparison of motion correction techniques applied to real cognitive data. Neuroimage 85, 181–191. 10.1016/j.neuroimage.2013.04.082 23639260 PMC3762942

[B6] Cui L. Chen G. Xu Z. Liu L. Wang H. Guo L.. (2017). Cerebral blood flow and its connectivity features of auditory verbal hallucinations in schizophrenia: A perfusion study. Psychiatry Res. Neuroimaging. 260, 53–61. 10.1016/j.pscychresns.2016.12.006 28024236

[B7] Curčić-Blake B. Ford J. Hubl D. Orlov N. Sommer I. Waters F.. (2017). Interaction of language, auditory and memory brain networks in auditory verbal hallucinations. Prog. Neurobiol. 148, 1–20. 10.1016/j.pneurobio.2016.11.002 27890810 PMC5240789

[B8] DeLisi L. (2001). Speech disorder in schizophrenia: review of the literature and exploration of its relation to the uniquely human capacity for language. Schizophr. Bull. 27, 481–496. 10.1093/oxfordjournals.schbul.a006889 11596849

[B9] Dugré J. Guay J. Dumais A. (2018). Risk factors of compliance with self-harm command hallucinations in individuals with affective and non-affective psychosis. Schizophr. Res. 195, 115–121. 10.1016/j.schres.2017.09.001 28911915

[B10] Ehlis A.-C. Schneider S. Dresler T. Fallgatter A. J. (2014). Application of functional near-infrared spectroscopy in psychiatry. NeuroImage 85, 478–488. 10.1016/j.neuroimage.2013.03.067 23578578

[B11] Feinberg I. (1978). Efference copy and corollary discharge: implications for thinking and its disorders. Schizophr. Bull. 4, 636–640. 10.1093/schbul/4.4.636 734369

[B12] Ferrari M. Quaresima V. (2012). A brief review on the history of human functional near-infrared spectroscopy (fNIRS) development and fields of application. NeuroImage. 63, 921–935. 10.1016/j.neuroimage.2012.03.049 22510258

[B13] García-Martí G. Aguilar E. Lull J. Martí-Bonmat,í L. Escart,í M. Manjón J.. (2008). Schizophrenia with auditory hallucinations: a voxel-based morphometry study. Prog. Neuropsychopharmacol. Biol. Psychiatry. 32, 72–80. 10.1016/j.pnpbp.2007.07.014 17716795

[B14] Gsell W. De Sadeleer C. Marchalant Y. MacKenzie E. Schumann P. Dauphin F. (2000). The use of cerebral blood flow as an index of neuronal activity in functional neuroimaging: experimental and pathophysiological considerations. J. Chem. Neuroanat. 20, 215–224. 10.1016/S0891-0618(00)00095-8 11207420

[B15] Haddock G. McCarron J. Tarrier N. Faragher E. B. (1999). Scales to measure dimensions of hallucinations and delusions: the psychotic symptom rating scales (PSYRATS). Psychol. Med. 29, 879–889. 10.1017/S0033291799008661 10473315

[B16] Herrmann M. J. Ehlis A. C. Fallgatter A. J. (2003). Frontal activation during a verbal-fluency task as measured by near-infrared spectroscopy. Brain Res. Bull. 61, 51–56. 10.1016/S0361-9230(03)00066-2 12788206

[B17] Jang K. Tak S. Jung J. Jang J. Jeong Y. Ye J. (2009). Wavelet minimum description length detrending for near-infrared spectroscopy. J. Biomed. Opt. 14, 034004. 10.1117/1.3127204 19566297

[B18] Jardri R. Larøi F. Waters F. (2019). Hallucination research: into the future, and beyond. Schizophr. Bull. 45, S1–S4. 10.1093/schbul/sby170 30715538 PMC6357977

[B19] Jardri R. Pouchet A. Pins D. Thomas P. (2011). Cortical activations during auditory verbal hallucinations in schizophrenia: a coordinate-based meta-analysis. Am. J. Psychiatry. 168, 73–81. 10.1176/appi.ajp.2010.09101522 20952459

[B20] Jones S. (2010). Do we need multiple models of auditory verbal hallucinations? Examining the phenomenological fit of cognitive and neurological models. Schizophr. Bull. 36, 566–575. 10.1093/schbul/sbn129 18820262 PMC2879699

[B21] Joo S. Yoon W. Jo Y. Kim H. Kim Y. Lee J. (2020). Aberrant executive control and auditory networks in recent-onset schizophrenia. Neuropsychiatr. Dis. Treat. 16, 1561–1570. 10.2147/NDT.S254208 32606708 PMC7319504

[B22] Kennedy J. Altar C. Taylor D. Degtiar I. Hornberger J. (2014). The social and economic burden of treatment-resistant schizophrenia: a systematic literature review. Int. Clin. Psychopharmacol. 29, 63–76. 10.1097/YIC.0b013e32836508e6 23995856

[B23] Koike S. Nishimura Y. Takizawa R. Yahata N. Kasai K. (2013). Near-infrared spectroscopy in schizophrenia: a possible biomarker for predicting clinical outcome and treatment response. Front. Psychiatry. 4, 145. 10.3389/fpsyt.2013.00145 24294205 PMC3827961

[B24] Koike S. Satomura Y. Kawasaki S. Nishimura Y. Kinoshita A. Sakurada H.. (2017). Application of functional near infrared spectroscopy as supplementary examination for diagnosis of clinical stages of psychosis spectrum. Psychiatry Clin. Neurosci. 71, 794–806. 10.1111/pcn.12551 28692185

[B25] Kühn S. Gallinat J. (2012). Quantitative meta-analysis on state and trait aspects of auditory verbal hallucinations in schizophrenia. Schizophr. Bull. 38, 779–786. 10.1093/schbul/sbq152 21177743 PMC3406531

[B26] Kumar V. Shivakumar V. Chhabra H. Bose A. Venkatasubramanian G. Gangadhar B. (2017). Functional near infra-red spectroscopy (fNIRS) in schizophrenia: a review. Asian J. Psychiatr. 27, 18–31. 10.1016/j.ajp.2017.02.009 28558892

[B27] Langland-Hassan P. (2010). Fractured phenomenologies: thought insertion, inner speech, and the puzzle of extraneity. Mind Languag. 23, 369–401. 10.1111/j.1468-0017.2008.00348.x

[B28] Lee W. Kennedy N. Bikson M. Frangou S. (2018). A Computational Assessment of Target Engagement in the Treatment of Auditory Hallucinations with Transcranial Direct Current Stimulation. Front. Psychiatry. 9, 48. 10.3389/fpsyt.2018.00048 29520240 PMC5826940

[B29] Lewine R. Fogg L. Meltzer H. (1983). Assessment of negative and positive symptoms in schizophrenia. Schizophr. Bull. 9, 368–376. 10.1093/schbul/9.3.368 6622994

[B30] McCarthy-Jones S. Resnick P. (2014). Listening to voices: the use of phenomenology to differentiate malingered from genuine auditory verbal hallucinations. Int. J. Law Psychiatry. 37, 183–189. 10.1016/j.ijlp.2013.11.004 24268827

[B31] McGuire P. Silbersweig D. Wright I. Murray R. David A. Frackowiak R.. (1995). Abnormal monitoring of inner speech: a physiological basis for auditory hallucinations. Lancet. 346, 596–600. 10.1016/S0140-6736(95)91435-8 7651003

[B32] McGuire P. Silbersweig D. Wright I. Murray R. Frackowiak R. Frith C. (1996). The neural correlates of inner speech and auditory verbal imagery in schizophrenia: relationship to auditory verbal hallucinations. Br. J. Psychiatry. 169, 148–159. 10.1192/bjp.169.2.148 8871790

[B33] Moseley P. Fernyhough C. Ellison A. (2013). Auditory verbal hallucinations as atypical inner speech monitoring, and the potential of neurostimulation as a treatment option. Neurosci. Biobehav. Rev. 37, 2794–2805. 10.1016/j.neubiorev.2013.10.001 24125858 PMC3870271

[B34] Narita Z. Noda T. Setoyama S. Sueyoshi K. Inagawa T. Sumiyoshi T. (2018). The effect of transcranial direct current stimulation on psychotic symptoms of schizophrenia is associated with oxy-hemoglobin concentrations in the brain as measured by near-infrared spectroscopy: a pilot study. J. Psychiatr. Res. 103, 5–9. 10.1016/j.jpsychires.2018.05.004 29754106

[B35] Nenadic I. Smesny S. Schlösser R. Sauer H. Gaser C. (2010). Auditory hallucinations and brain structure in schizophrenia: voxel-based morphometric study. Br. J. Psychiatry. 196. 412–413. 10.1192/bjp.bp.109.070441 20435970

[B36] Okada N. Takahasi K. Nishimura Y. Koike S. Ishii-Takahashi A. Sakakibara E.. (2016). Characterizing prefrontal cortical activity during inhibition task in methamphetamine-associated psychosis versus schizophrenia: a multi-channel near-infrared spectroscopy study. Addict. Biol. 21, 489–503. 10.1111/adb.12224 25619621

[B37] Pérez-Bellido A. Anne Barnes K. Crommett L. Yau J. (2018). Auditory frequency representations in human somatosensory cortex. Cereb. Cortex. 28, 3908–3921. 10.1093/cercor/bhx255 29045579 PMC6188539

[B38] Raij T. Riekki T. (2012). Poor supplementary motor area activation differentiates auditory verbal hallucination from imagining the hallucination. NeuroImage Clin. 1, 75–80. 10.1016/j.nicl.2012.09.007 24179739 PMC3757718

[B39] Ratcliff K. Farhall J. Shawyer F. (2011). Auditory hallucinations: a review of assessment tools. Clin. Psychol. Psychother. 18, 524–534. 10.1002/cpp.729 22131297

[B40] Sasai S. Homae F. Watanabe H. Sasaki A. Tanabe H. Sadato N.. (2012). A NIRS-fMRI study of resting state network. NeuroImage. 63, 179–193. 10.1016/j.neuroimage.2012.06.011 22713670

[B41] Shergill S. Brammer M. Fukuda R. Bullmore E. Amaro E. Murray R.. (2002). Modulation of activity in temporal cortex during generation of inner speech. Hum. Brain Mapp. 16, 219–227. 10.1002/hbm.10046 12112764 PMC6871832

[B42] Shergill S. S. (2003). Engagement of brain areas implicated in processing inner speech in people with auditory hallucinations. Br. J. Psychiatry 182, 525–531. 10.1192/bjp.182.6.525 12777344

[B43] Simons C. Tracy D. Sanghera K. O'Daly O. Gilleen J. Dominguez M.. (2010). Functional magnetic resonance imaging of inner speech in schizophrenia. Biol. Psychiatry. 67, 232–237. 10.1016/j.biopsych.2009.09.007 19846064

[B44] Simons J. Davis S. Gilbert S. Frith C. Burgess P. (2006). Discriminating imagined from perceived information engages brain areas implicated in schizophrenia. NeuroImage. 32, 696–703. 10.1016/j.neuroimage.2006.04.209 16797186

[B45] Singh A. K. Dan I. (2006). Exploring the false discovery rate in multichannel NIRS. Neuroimage 33, 542–549. 10.1016/j.neuroimage.2006.06.047 16959498

[B46] Skipper J. Nusbaum H. Small S. (2005). Listening to talking faces: motor cortical activation during speech perception. NeuroImage. 25, 76–89. 10.1016/j.neuroimage.2004.11.006 15734345

[B47] Stephane M. Barton S. Boutros N. N. (2001). Auditory verbal hallucinations and dysfunction of the neural substrates of speech. Schizophr. Res. 50, 61–78. 10.1016/s0920-9964(00)00150-x 11378315

[B48] Strangman G. Franceschini M. Boas D. (2003). Factors affecting the accuracy of near-infrared spectroscopy concentration calculations for focal changes in oxygenation parameters. NeuroImage. 18, 865–879. 10.1016/S1053-8119(03)00021-1 12725763

[B49] Suto T. Fukuda M. Ito M. Uehara T. Mikuni M. (2004). Multichannel near-infrared spectroscopy in depression and schizophrenia: cognitive brain activation study. Biol. Psychiatry. 55, 501–511. 10.1016/j.biopsych.2003.09.008 15023578

[B50] Takizawa R. Fukuda M. Kawasaki S. Kasai K. Mimura M. Pu S.. (2014). Neuroimaging-aided differential diagnosis of the depressive state. NeuroImage. 85, 498–507. 10.1016/j.neuroimage.2013.05.126 23764293

[B51] Takizawa R. Kasai K. Kawakubo Y. Marumo K. Kawasaki S. Yamasue H.. (2008). Reduced frontopolar activation during verbal fluency task in schizophrenia: a multi-channel near-infrared spectroscopy study. Schizophr. Res. 99, 250–262. 10.1016/j.schres.2007.10.025 18063344

[B52] Tremblay S. Shiller D. Ostry D. (2003). Somatosensory basis of speech production. Nature 423, 866–869. 10.1038/nature01710 12815431

[B53] Upthegrove R. Broome M. Caldwell K. Ives J. Oyebode F. Wood S. (2016). Understanding auditory verbal hallucinations: a systematic review of current evidence. Acta Psychiatr. Scand. 133, 352–367. 10.1111/acps.12531 26661730

[B54] van Lutterveld R. Diederen K. Koops S. Begemann M. Sommer I. (2013). The influence of stimulus detection on activation patterns during auditory hallucinations. Schizophr. Res. 145, 27–32. 10.1016/j.schres.2013.01.004 23375942

[B55] Waters F. (2012). Multidisciplinary approaches to understanding auditory hallucinations in schizophrenia and nonschizophrenia populations: the International Consortium on Hallucination Research. Schizophr. Bull. 38, 693–694. 10.1093/schbul/sbs070 22837351 PMC3406516

[B56] Wayne W. U. (2012). Explaining schizophrenia: auditory verbal hallucination and self-monitoring. Mind Languag. 27, 86–107. 10.1111/j.1468-0017.2011.01436.x

[B57] Wei Y. Chen Q. Curtin A. Tu L. Tang X. Tang Y.. (2020). Functional near-infrared spectroscopy (fNIRS) as a tool to assist the diagnosis of major psychiatric disorders in a Chinese population. Eur. Arch. Psychiatry Clin. Neurosci. 271, 745–757. 10.1007/s00406-020-01125-y 32279143

[B58] Ye J. Tak S. Jang K. Jung J. Jang J. (2009). NIRS-SPM: statistical parametric mapping for near-infrared spectroscopy. NeuroImage. 44, 428–447. 10.1016/j.neuroimage.2008.08.036 18848897

[B59] Zhuo C. Zhu J. Wang C. Wang L. Li J. Qin W. (2016). Increased local spontaneous neural activity in the left precuneus specific to auditory verbal hallucinations of schizophrenia. Chin. Med. J. 129, 809–813. 10.4103/0366-6999.178974 26996476 PMC4819301

